# Serum-therapeutics1Read before the Pennsylvania State Veterinary Medical Association at Cresson Springs, September 3, 1895. The experimental information is drawn from Annales de Pasteur Institute, September, 1894.

**Published:** 1896-01

**Authors:** S. J. J. Harger


### SERUM-THERAPEUTICS.[1]

By S. J. J. HARGER, V.M.D.

The discovery of serum-therapeutics, now applied in practice, has opened a new era in the treatment of disease. Although confined as yet to a limited number of diseases, and not yet thoroughly tested, this method of treatment has opened a field of experimentation whose limits to-day we cannot define. A general *résumé* of the subject may not be amiss before this meeting.

It is known that the vegetable alkaloids, abrin, ricin, etc., very poisonous to animals, can, when given in repeated and very small increasing doses, develop an immunity in these animals enabling them to withstand a quantity of these substances rapidly fatal to those not previously treated. A product is formed in the system which becomes sufficiently abundant to neutralize the toxic action of these poisons. Likewise in morbid processes, the repeated introduction into the system of a microbe or its product, called *toxin*, causes the formation in the economy of a substance called *antitoxin*, which is capable of counteracting the pathogenic action of such microbes or their poisonous excretions. This, in a word, is the basis of the so-called antitoxin treatment. The antitoxin thus successfully combats, reciprocally, the microbes and their products to which it owes its origin, at least indirectly.

Serum-therapeutics was first studied in a crude way in cowpox and septicæmia in the dog, but has only attracted special attention since Behring and Kitasato have demonstrated the properties of the blood-serum of animals immunized against tetanus and diphtheria. They recognized in the blood-serum

[1] Read before the Pennsylvania State Veterinary Medical Association at Cresson Springs, September 3, 1895. The experimental information is drawn from Annales de Pasteur Institute, September, 1894.

of such animals both preventive and curative properties when injected either before or after the introduction of disease germs into the body.

Some antitoxin blood-serums have the power of counteracting the virus of other diseases. Thus, the serum of animals immunized against symptomatic anthrax is effective against the bacillus of acute septicæmia (Duntschman). The serum of a healthy man, and at times that of the horse, is immunizing against the intraperitoneal injections of cholera (Pfeiffer). The prophylactic action of blood-serum is therefore not absolutely specific; it is possessed in some instances by normal serum.

Both the toxin and antitoxin are albuminous bodies, very similar in chemical properties, but different in physiological action and precipitated by alcohol, which enables its preparation in a condensed form.

*Preparation of the toxin.* The process of preparing the toxin varies with the variety of the specific microbe. Suffice it to say that the latter is grown upon a favorable medium, such as bouillon, gelatin, agar, etc., the mature culture (diphtheria) being passed through a Chamberland filter; the filtrate, a clear liquid containing the toxin, is preserved in well-filled, corked bottles. It gradually loses its activity.

*Immunization of the animal.* This may be accomplished by subcutaneous injections of small repeated doses of pure or heated toxin; a better method, that of Roux and Villard, consists in mixing three parts of toxin with one of Gram's solution of iodine. The initial dose in the rabbit is ½ c.c. (7 drops) repeated every three or four days for several weeks; the dose is then gradually increased, and finally undiluted toxin is given. The horse is usually the animal selected to furnish the antitoxin serum of tetanus and diphtheritic virus, but the serum can be obtained in large quantities by bleeding from the jugular vein. and, besides, this serum has proved to have a strong antitoxic power. Of course he must be free from any disease, and glanders must be eliminated by test-injection of mallein. For diphtheria the cow, dog, sheep, goat, and rabbit can also be used. The cow is very sensitive to the diphtheritic poison, even fatally so; the toxin is excreted with the milk.

*Formation of the antitoxin.* The antitoxin is a cellular secretion, but it is not determined which set of cells are active in this process. It is not a transformation in the body of the toxin injected. The antitoxin diminishes with the cessation of the toxin injections. In one experiment, two rabbits were each injected with 103 c.c. of tetanus toxin, the first in 33 daily doses and the second in 9 larger doses. The serum of the first neutralized 150 parts of tetanus toxin; that of the latter only 25 parts. The two serums were of unequal preventive power; the quantity of toxin injected was the same in both cases, but the methods of administration differed. By successive bleedings in the rabbit a quantity of blood equivalent to the entire volume of the circulation can be removed, and the antitoxic power of the blood is produced as rapidly as it is drawn away without any new injections of toxin.

An important question is the manner of action of the antitoxin after introduction into the body. In diphtheria and tetanus it counteracts the effects of the toxin; in some diseases it is protective only against the microbe and such animals as remain as sensitive to the toxin as those which are not immunized.

A mixture of antitoxin with an immunizing power of one billion and tetanus-toxin 1/1000 c.c. of which destroys a mouse, in proportion of 1-900, becomes harmless. One-half c.c. (1/1800 c.c. serum) injected into a guinea-pig does not produce tetanus. This result, however, is not always the same; a mixture innocuous to mice is fatal to guinea-pigs. In eight out of ten guinea-pigs ½ c.c. (1–900) is inactive, while the remaining two develop tetanus; again, 1 c.c. (1–500) is harmless to guinea-pigs, while 3 c.c. cause tetanus.

These experiments show that it is not a question of neutralization or chemical union between the toxin and antitoxin in the blood, but rather the stimulating action of the latter upon the animal cells, enabling them to resist the action of the virus of the disease.

The existence of a previous disease or a previous injection of microbic products is not without effect. It weakens the animal tissues, which respond less strongly to the stimulating action of the antitoxin. Roux and Villard injected five guinea-pigs with ½ c.c. of antitoxin-serum and toxin (1–900) with a negative result. Five other guinea-pigs, which had some time before been immuned against the vibrion of Massaonah, developed tetanus, with the same treatment as the preceding five. One-third c.c. only produced tetanus when the animal was subsequently injected with microbic product, coli bacilli, etc.

In eighty days this horse (the details of immunization of which I will cite at the end of this paper) received over 800 c.c.

of toxin without any symptoms other than a slight local œdema and an elevation of temperature of one degree.

The serum of a horse immunized in this manner had a preventive power of more than 50,000; that is, a guinea-pig inoculated with 1/50,000 part of its own weight of the serum twelve hours before will resist the action of ½ c.c. of a virulent culture of diphtheria bacillus. One-tenth c.c. of this serum and 1 c.c. of diphtheria toxin do not produce any œdema in the guinea-pig when injected under the skin. The horse is kept in this condition of immunization by injections at intervals of fifteen to twenty days of diphtheritic toxin (about 200 to 250 c.c.). Roux and Martin immunized the horse with 40 c.c. of the toxin, heated to 65° C., given in eight doses at intervals of ten days; he was then injected with 1 c.c. of pure toxin repeated at regular intervals. The virulent bacilli are also employed for this purpose. The serum employed by Roux and Martin in children and guinea-pigs in doses of 1/10 c.c. killed the latter in forty-eight hours. Guinea-pigs injected with a quantity of serum equal to 1/100,000 part of their weight resisted a dose fatal to control-animals in five days; 1/50 000 immunized them against a quantity fatal in forty-eight hours.

Infection having taken place, the quantity of serum necessary to preserve life must be in proportion to the time elapsed since the introduction of the poison into the system. Guinea-pigs receiving a dose fatal in forty-eight hours survived after an injection equal to 1/1000 part of their weight six hours afterward; the same treatment twelve hours afterward was ineffective. This is the principle of the serum-treatment of diphtheria in children which in many instances has given such happy results. The antitoxin is not of uniform strength in all cases, a fact which explains the alarming and even fatal symptoms occasionally seen. The dose must be graduated according to the virulency of the serum, the weight of the subject, and the time elapsed since the introduction of the poison.

The following illustration will show the manner of immunization: Horse, seven years of age; weight 900 pounds. Serum very active; 1/10 c.c. killed guinea-pig in forty-eight hours. Injected hypodermically in the neck or behind the shoulder.

1*st day.* Injection, ¼ c.c. toxin (1/10 iodine); no reaction, local or general.

2*d.* Injection, ½ c.c. toxin (1/10 iodine).

4*th*, 6*th, and* 8*th.* Injection, ½ c.c. toxin (1/10 iodine).

13*th*, 14*th*. Injection, 1 c.c. toxin (1/10 iodine); no reaction.

17*th*. Injection, ¼ c.c. pure toxin; slight œdema, no fever.

22*d*. Injection, 1 c.c. pure toxin; slight œdema, no fever.

23*d*. Injection, 2 c.c. pure toxin; slight œdema, no fever.

25*th*. Injection, 3 c.c pure toxin; slight œdema, no fever.

28*th*. Injection, 5 c.c. pure toxin; slight œdema, no fever.

30*th*, 32*d*, 36*th*. Injection, 5 c.c. pure toxin; slight œdema, no fever.

39*th*, 41*st*. Injection, 10 c.c. pure toxin; slight œdema, no fever.

43*d*, 46*th*, 48*th*, 50*th*. Injection, 30 c.c. pure toxin; œdema marked, disappearing in forty-eight hours.

53*d*. Injection, 60 c.c. pure toxin; œdema marked, disappearing in forty-eight hours.

57*th*, 63*d*, 65*th*, 67*th*. Injection, 60 c.c. pure toxin; œdema marked, disappearing in forty-eight hours.

72*d*. Injection, 90 c.c. pure toxin; œdema marked, disappearing in forty-eight hours.

80*th*. Injection, 250 c.c. pure toxin; œdema marked, disappearing in forty-eight hours.

TETANUS. The toxin of *tetanus* is very active. A healthy horse has been destroyed by 1/10 c.c. (2 drops). Horses can be immunized against tetanus in the same manner as against diphtheria. The toxin, pure, heated to 65° C., or mixed with iodine, is injected in repeated and gradually increasing doses at intervals. The animal thus becomes so "accustomed" to the poison that 250 to 300 c.c. can be given at a single injection, enough to destroy 2500 horses (Nocard).

This serum is preventive and curative; it can be used as a vaccine or a therapeutic agent. Injected under the skin of a tetanic subject, it is efficacious only in proportion as the time elapsed since the infection has been short. I have no statistics of this treatment in the horse, but hope to be able to give it a trial. It is useful as a preventive only when the virus, or shortly afterward, is introduced into the circulation. Unlike diphtheria, tetanus is not recognized until the system is saturated with the poison, then no treatment is of much avail.

The serum may be recommended as a preventive in places where tetanus is almost epizoötic, or in certain operations and wounds of the extremities which are frequently followed by tetanus, in addition to the regular surgical treatment. For this purpose Nocard injects under the skin behind the shoulder 10 c.c. of antitoxin-serum, repeated in two weeks.

Let me suggest peroxide of hydrogen as a local dressing in wounds of the extremities in particular. This preparation freely gives off oxygen, and the tetanus bacillus, which is confined to the seat of traumatism and does not enter the circulation, cannot live in a medium exposed to oxygen.

